# TrkB signaling is correlated with muscular fatigue resistance and less vulnerability to neurodegeneration

**DOI:** 10.3389/fnmol.2022.1069940

**Published:** 2022-12-22

**Authors:** Laia Just-Borràs, Víctor Cilleros-Mañé, Aleksandra Polishchuk, Marta Balanyà-Segura, Marta Tomàs, Neus Garcia, Josep Tomàs, Maria A. Lanuza

**Affiliations:** Unitat d’Histologia i Neurobiologia, Departament de Ciències Mèdiques Bàsiques, Facultat de Medicina i Ciències de la Salut, Universitat Rovira i Virgili, Reus, Spain

**Keywords:** TrkB-BDNF, PKC, PKA, neuromuscular junction, skeletal muscle, EOM, fatigue-resistant

## Abstract

At the neuromuscular junction (NMJ), motor neurons and myocytes maintain a bidirectional communication that guarantees adequate functionality. Thus, motor neurons’ firing pattern, which is influenced by retrograde muscle-derived neurotrophic factors, modulates myocyte contractibility. Myocytes can be fast-twitch fibers and become easily fatigued or slow-twitch fibers and resistant to fatigue. Extraocular muscles (EOM) show mixed properties that guarantee fast contraction speed and resistance to fatigue and the degeneration caused by Amyotrophic lateral sclerosis (ALS) disease. The TrkB signaling is an activity-dependent pathway implicated in the NMJ well-functioning. Therefore, it could mediate the differences between fast and slow myocytes’ resistance to fatigue. The present study elucidates a specific protein expression profile concerning the TrkB signaling that correlates with higher resistance to fatigue and better neuroprotective capacity through time. The results unveil that Extra-ocular muscles (EOM) express lower levels of NT-4 that extend TrkB signaling, differential PKC expression, and a higher abundance of phosphorylated synaptic proteins that correlate with continuous neurotransmission requirements. Furthermore, common molecular features between EOM and slow soleus muscles including higher neurotrophic consumption and classic and novel PKC isoforms balance correlate with better preservation of these two muscles in ALS. Altogether, higher resistance of Soleus and EOM to fatigue and ALS seems to be associated with specific protein levels concerning the TrkB neurotrophic signaling.

## 1 Introduction

Motor neurons (MNs) interact with myocytes through the neuromuscular junction (NMJ) to guarantee their adequate functionality in a feedback loop mode through the neuromotor and neurotrophic control mechanisms. In brief, MNs regulate the properties of the myocytes they innervate through their firing activity pattern, determined by their intrinsic properties and by the inputs that they receive. In their turn, myocytes influence MNs through the retrograde activity-dependent secretion of neurotrophic factors (Baldwin et al., 2013; Cisterna et al., 2014; Hurtado et al., 2017b).

One MN innervates several myocytes to form a motor unit (MU). At their time, several MU innervate all the myocytes in one muscle to generate a complete functional contraction. Consequently, fast and slow skeletal muscles are differently innervated and combine their intrinsic characteristics to organize the functionality of a whole limb. MNs are named fast-twitch fatigable (FF), fast-twitch fatigue-resistant (FR), and slow-twitch fatigue-resistant (S) in accordance with the properties of the myocytes they innervate (Kanning et al., 2010). In their turn, myocytes are classified by their contraction speed and strength, which depend on myosin heavy chain expression (Ausoni et al., 1990) and mitochondrial activity, which is higher in slow-twitching fibers (Leary et al., 2003; Hakamata et al., 2018). Slow-twitching muscles present a more oxidative and fatigue-resistant metabolism than fast-twitching ones, which are preferentially glycolytic (Delp and Duan, 1996; Schiaffino and Reggiani, 2011). Finally, extraocular muscles (EOM), which are among the fastest muscles in mammals, have unique characteristics, including the expression of specific MHC isoforms to guarantee both fast contraction speed and vast resistance to fatigue (Porter et al., 1995). Furthermore, EOMs are less vulnerable to Amyotrophic lateral sclerosis (ALS) neurodegenerative disease, like slow and fatigue-resistant muscles.

Among the muscles used in the present study, Extensor Digitorum Longus (EDL) and Tibialis Anterior (TA) are fast extensor and flexor muscles, respectively, mainly composed of fast-twitching fibers. On the other hand, Soleus (SOL) is composed of slow-twitching fibers (Novák et al., 2010). Type I and IIA NMJ nerve terminals are smaller and less branched (Mech et al., 2020). Also, they have deeper postsynaptic gutters and a more efficient synaptic vesicle recycling mechanism in comparison with fiber IIB (Tomas et al., 1992; Mantilla et al., 2007), which is consistent with an enlarged pool of releasable synaptic vesicles found in the slower fibers (Reid et al., 1999). Indeed, the EDL vesicle pool is ∼33% smaller than SOL one despite that recycling times are equal (Reid et al., 1999). Consequently, EDL’s ability to sustain neuromuscular transmission during repeated activation is minor (Miyata et al., 1995; Prakash et al., 1999). Therefore, fiber composition in EDL is prepared to support relatively short bursts of strong contractions and it is easily fatigued after a short time of activity. In contrast, SOL is resistant to fatigue as MN are able to release ACh for extended periods of time at a lower frequency and myocytes smoothly contract in response. During stimulation, the initial quantal content to generate the end-plate potentials (EPPs) is significantly higher in EDL than in SOL (Gertler and Robbins, 1978; Wood and Slater, 2001) while EPP amplitude further decreases in EDL than in SOL (Reid et al., 1999). However, under continuous stimulation, motor units are able to adapt from faster to slower activity patterns and gain resistance to fatigue, both molecularly (Just-Borràs et al., 2021) and functionally (Windisch et al., 1998). Altogether resistance to fatigue during tonic stimulation is mainly sustained by larger vesicle pools and lower quantal content in which SNARE-SM proteins are involved. In opposition to limb muscles, EOMs combine fast, slow, embryonic, and EOM-specific MHC isoforms to guarantee fast contraction speed and vast resistance to fatigue (Zhou et al., 2011).

The Brain-derived neurotrophic factor (BDNF)/Tropomyosin receptor kinase B (TrkB) signaling is a retrograde neurotrophic signaling tightly related to NMJ stability and neurotransmission. In brief, nerve-induced muscle contraction regulates the pathway to retrogradely modulate the activity of presynaptic protein kinases PKC and PKA on SNARE-SM targets (Hurtado et al., 2017a,b; Simó et al., 2018, 2019), directly involved in the control of the ACh release. Consequently, TrkB regulates both the postsynaptic and presynaptic components to adapt the neurotransmission mechanism to the requirements of each synapse (Lu, 2003; Mantilla et al., 2004, 2014; Matthews et al., 2009; Garcia et al., 2010a; Santafé et al., 2014; Hurtado et al., 2017a,b; Simó et al., 2018, 2019).

**FIGURE 1:**
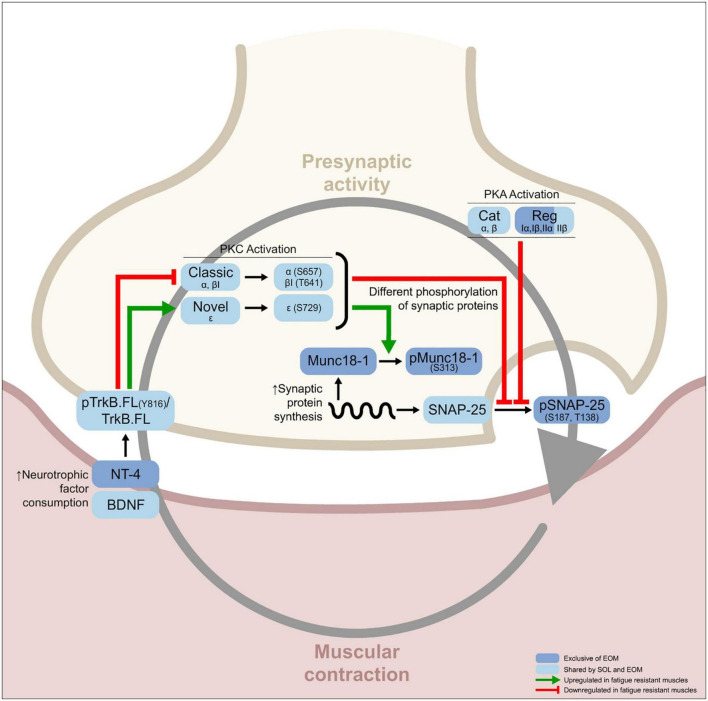
Graphical abstract showing the differential molecular features of fatigue resistant muscles [soleus (SOL) and extraocular muscles (EOM)] in comparison with fatigable muscles [extensor digitorum longus (EDL) and tibialis anterior (TA)]. Following the circular relation between the presynaptic and postsynaptic components of the neuromuscular junction (NMJ) gray arrow characterized by previous work done in Sprague Dawley rat diaphragms stimulated through the phrenic nerve *ex vivo* and activating or inhibiting each one of the proteins in the pathway (Obis et al., 2015a,b; Hurtado et al., 2017a,b; Simó et al., 2018, 2019; Cilleros-Mañé et al., 2020, 2021), one can identify that muscular resistance to fatigue is mediated by constant neurotrophic consumption that modulates tropomyosin receptor kinase B (TrkB) phosphorylation ratio. Consequently, PKC activity in both SOL and EOM muscles is modified, which results in a differential balance of synaptic protein phosphorylation between fatigable and fatigue resistant muscles. Also, PKA activity and synaptic protein abundance are differential between fatigue resistant and fatigable muscles. Many protein levels are shared between SOL and EOM, indicated in pale blue squares. However, there are specific differences between the two fatigue resistant muscles SOL and EOM indicated in dark blue squares.

Here, we hypothesize that BDNF signaling pathway expression and activity should be different in slow and fast limb muscles and in the EOMs, in accordance with their activity patterns. Furthermore, it could have an impact on their resistance to fatigue and the ALS effect. Indeed, Delezie et al. (2019) reported that reducing BDNF expression in fast muscles was correlated with a slower metabolism acquisition in fast muscles and neurturin expression, which is related to the capillary density and oxidative capacity, was also related to slower action patterns (Correia et al., 2021). Furthermore, previous works reported that the TrkB pathway was disturbed in fast muscles affected by ALS (Just-Borràs et al., 2019; Tosolini et al., 2022), while exercise, that improved mice phenotype (Deforges et al., 2009), reduced the molecular alterations (Just-Borràs et al., 2019, 2020). Also, BDNF administration reduced the endosome traffic reduction *in vivo* in SOD1-G93A mice (Tosolini et al., 2022). Thus, the higher resistance to ALS neurodegenerative disease of the EOM and the slow limb soleus muscle could be associated with well-adapted BDNF/TrkB neurotrophic signaling. Consequently, understanding how the TrkB pathway works under physiological conditions in different skeletal muscles will help to understand the preferential affection of the myocytes in relation to different neuroprotection capacities. Altogether the present study compares the molecular phenotype of the TrkB signaling pathway in two fast and one slow limb muscles and EOM to find the different protein expressions of these last ones that could result in their specific resistance to fatigue and disease. In brief, the results of this article elucidate the relation between differential protein expression in each muscle and its physiological demands. Thus, EOMs express lower levels of NT-4 that extend TrkB signaling through time and a higher abundance of phosphorylated synaptic proteins, that correlate with continuous synaptic activity. Furthermore, common features between EOM and soleus muscles, including classic and novel PKC isoforms balance, also correlate with better preservation of these two muscles in ALS (Figure 1).

## 2 Materials and methods

### 2.1 Animal care

Adult male Sprague Dawley rats (50 days of age; Criffa, Barcelona, Spain; RRID: RGD_5508397) were kept in the animal facility under standard conditions: constant temperature (22 ± 2^°^C), relative humidity (50 ± 10%), a 12-h light/dark schedule and unrestricted access to food and water. Experiments were performed under the approval of the Ethics Committee of Animal Experimentation of the Universitat Rovira i Virgili, in accordance with the European Union Directive 2010/63/EU guidelines for the humane treatment of laboratory animals. Muscles were obtained from six animals from the colony (*n* = 6) to be used as biological replicates.

### 2.2 Western blotting

Animals were euthanized and immediately afterward Extensor Digitorum Longs (EDL), Tibialis Anterior (TA), Soleus (SOL), and the four rectus EOM muscles were dissected from their insertions and frozen in liquid nitrogen. Homogenization was performed with an overhead stirrer (VWR International, Clarksburg, MD) in ice-cold lysis buffer [in mM: NaCl 150, Tris–HCl 50 (pH 7.4), EDTA 1, NaF 50, PMSF 1, Na_3_VO_4_ 1; NP-40 1%, Triton X-100 0.1%, and protease inhibitor cocktail 1% (Sigma, Saint Louis, MO, USA)]. Insoluble materials were removed with two centrifugations at 4°C: 1,000 *g* for 10 min and 15,000 g for 20 min. The final supernatant contained the whole-cell fraction lysate. Protein concentrations were determined by DC protein assay (Bio-Rad, Hercules, CA, IL, USA).

The western blotting procedure was performed as previously described (Just-Borràs et al., 2019). Protein samples of 30 μg were separated by 8 or 12% SDS-polyacrylamide electrophoresis and electro-transferred to a polyvinylidene difluoride (PVDF) membrane (Hybond™-P; Amersham, GE Healthcare, Marlborough, MA, USA) using Trans-Blot Turbo Transfer System (Bio-Rad, Hercules, CA, USA). For immunodetection, membranes were blocked with TBST containing 5% (w/v) phosphoblocker or bovine serum albumin (BSA) for phosphorylated proteins and non-fat dry milk for non-phosphorylated proteins for an hour. Then, membranes were incubated in the primary antibody overnight and with the corresponding horseradish peroxidase-conjugated secondary antibody for 1 h (Table 1). Membranes were revealed with Bio-Rad ECL kid on the ChemiDoc XRS + machine (Bio-Rad, Hercules, CA, USA). The bands’ optical density was normalized in relation to (1) the background values and to (2) the total protein transferred on PVDF membranes, measured by total protein analysis [Sypro Ruby protein blot stain, Bio-Rad (Aldridge et al., 2008)]. The relative variations between samples were calculated from the same membrane image. Data were taken from densitometry measurements made in at least three biological replicates. Primary antibodies were omitted from some samples during the procedure as controls, and they never revealed bands of the expected molecular weight. Furthermore, their specificity has been proved in previous publications from the group (Obis et al., 2015b; Hurtado et al., 2017a,b; Simó et al., 2018, 2019; Cilleros-Mañé et al., 2020, 2021).

**TABLE 1:** List of primary and secondary antibodies used.

Target	Source	Reference	Dilution	Target	Source	Reference	Dilution
BDNF	Rb pAb	Sc-20981	1/500	PKA Cα	Rb pAb	Sc-903	1/1000
NT-4	Rb pAb	Sc-545	1/500	PKA Cβ	Rb pAb	Sc-904	1/1000
p75^NTR^	Rb pAb	07-476	1/800	PKA RIα	Ms mAb	Sc-136231	1/1000
TrkB	Ms mAb	Sc-377218	1/1000	PKA RIβ	Rb pAb	Sc-907	1/1000
pTrkB (Y816)	Rb pAb	ABN1381	1/1000	PKA RIIα	Rb pAb	Sc-909	1/1000
PDK1	Ms mAb	Sc-17765	1/1000	PKA RIIβ	Ms mAb	Sc-376778	1/1000
pDPK1 (S241)	Rb pAb	#3061	1/1000	Munc18-1	Rb mAb	13414	1/1000
cPKCα	Rb pAb	Sc-208	1/800	pMunc18-1 (S313)	Rb pAb	Ab138687	1/1000
pcPKCα (S657)	Rb pAb	06-822	1/1000	SNAP-25	Rb mAb	#5309	1/1000
cPKCβI	Rb pAb	Sc-209	1/1000	pSNAP-25 (S187)	Rb pAb	Ab169871	1/1000
pcPKCβI (T641)	Rb pAb	Ab75657	1/1000	pSNAP-25 (T138)	Rb pAb	Orb163730	1/1000
nPKCε	Rb pAb	Sc-214	1/1000	HRP-conjugated	Dk a-Rb pAb	711-035-152	1/10.000
pnPKCε (S729)	Rb pAb	Sc-12355	1/1000	HRP-conjugated	Rb a-Ms pAb	A9044	1/10.000

### 2.3 Statistical analysis

All values are represented as mean ± standard deviation (SEM) within each muscle. Also, each circle represents the value of one animal, normalized in relation to EDL muscle, whose value was transformed to 1, to visualize proportional data distribution. The statistical significance of the differences between the experimental groups was evaluated under a non-parametric Kruskal–Wallis test followed by Dunn’s *post-hoc* test (GraphPad Prism software, San Diego, CA, USA). The criterion for statistical significance was: *p < 0.05, ^**^p < 0.01, and ^***^p < 0.001.

## 3 Results

To determine the expression of the presynaptic TrkB neurotrophic signaling pathway that regulates synaptic vesicles exocytosis at the NMJ, we analyzed the entire TrkB pathway at the synapses of EDL, TA, SOL, and EOM muscles in its three molecular levels: (i) the neurotrophic factors BDNF and NT-4 and their receptors TrkB full length (TrkB.FL) and truncated (TrkB.T) and p75^NTR^, (ii) the coupled serine-threonine kinases C (PKC isoforms) and their priming kinase phosphoinositide-dependent kinase 1 (PDK1) and the different subunits of the cAMP-dependent kinase (PKA), and (iii) PKC and PKA targets related with ACh release (Munc18-1 and SNAP-25).

### 3.1 Neurotrophic factors and receptors expression is lower in fatigue-resistant muscles

**FIGURE 2:**
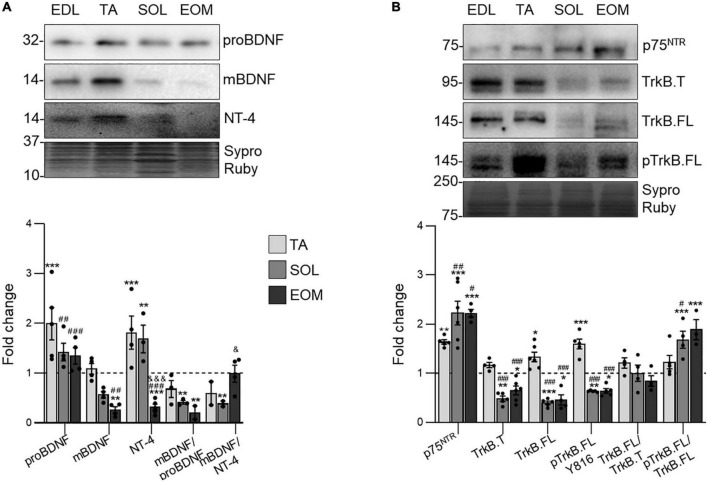
NTFs and receptors expression changes in EDL, TA, SOL, and EOM of Sprague Dawley rats. Western blot analysis and data quantification of **(A)** neurotrophic factors and **(B)** neurotrophic factor receptors protein levels in different muscles. Sypro Ruby images show the region surrounding each protein of interest despite the bands’ optical densities being normalized using the full length of the lanes. Data are presented as mean ± SEM. Each circle in the bars is the mean result of one animal. *P ≤ 0.05; **P ≤ 0.01; ***P ≤ 0.001; *against EDL, # against TA, & against SOL (Kruskal–Wallis test and Dunn’s *post-hoc* test). EDL, Extensor Digitorum Longus; TA, Tibialis Anterior; SOL, Soleus; EOM, Extraocular Muscles. Graphs done with GraphPad Prism software, San Diego, USA.

Figure 2A shows that proBDNF is uniformly expressed in 3 of the 4 studied muscles, being upregulated in TA. On the other hand, mature BDNF (mBDNF) decreases in SOL and EOM (Figure 2A). Consequently, the proportion between mature and pro BDNF detected (mBDNF/proBDNF ratio) is significantly lower in both EOM and SOL than in EDL and TA. On the other hand, NT-4 expression is significantly lower in EOM than in limb muscles.

Understanding neurotrophic factor consumption requires the analysis of the receptors that trigger their signaling. Thus, Figure 2B shows that p75^NTR^ expression is proportional to muscular fatigue resistance, being less expressed in EDL muscles and upregulated in SOL and EOM. On the other hand, TrkB is more expressed and phosphorylated in the fast, fatigable EDL and TA than in the fatigue resistant muscles SOL and EOM in healthy young adult Sprague Dawley rats. However, results reveal that TrkB.FL is proportionally more phosphorylated in SOL and EOM muscles, as the proportion between phosphorylated and total TrkB.FL detected (pTrkB.FL/TrkB.FL ratio) increases in these muscles. Furthermore, the proportion between full and truncated TrkB detected (TrkB.FL/TrkB.T) ratio is kept through all the studied muscles.

### 3.2 Classic and novel PKC isoforms differential expression determines muscle phenotype

**FIGURE 3:**
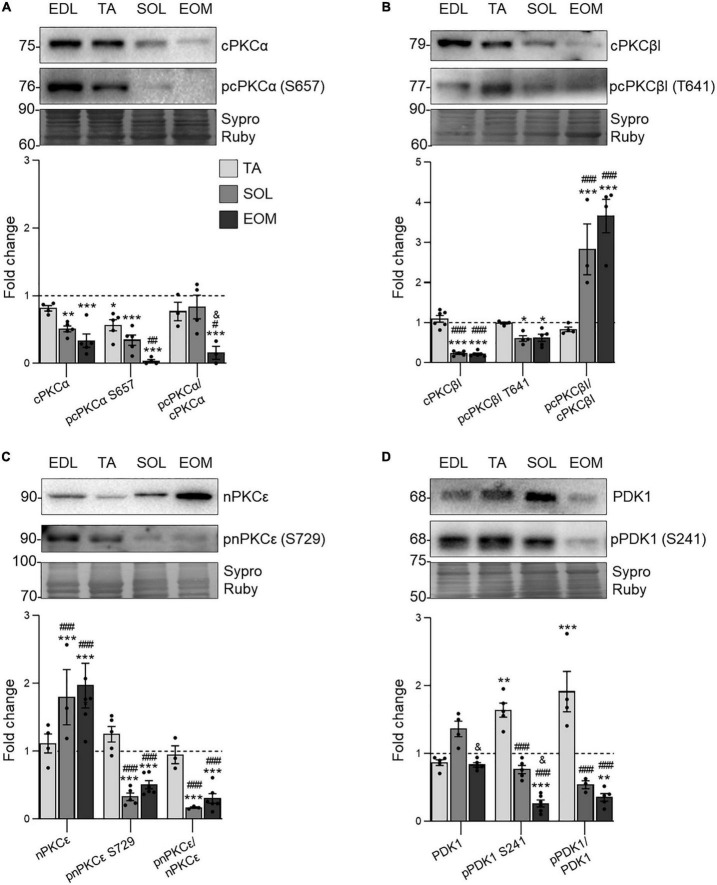
PKC isoforms and PDK1 expression changes in EDL, TA, SOL, and EOM of Sprague Dawley rats. Western blot analysis and data quantification of **(A)** PDK1, **(B)** cPKCα, **(C)** cPKCβI, and **(D)** nPKCε protein levels in different muscles. Sypro Ruby images show the region surrounding each protein of interest despite the bands’ optical densities being normalized using the full length of the lanes. Data are presented as mean ± SEM. Each circle in the bars is the mean result of one animal. *P ≤ 0.05; **P ≤ 0.01; ***P ≤ 0.001; *against EDL, # against TA, & against SOL (Kruskal–Wallis test and Dunn’s *post-hoc* test). EDL, Extensor Digitorum Longus; TA, Tibialis Anterior; SOL, Soleus; EOM, Extraocular Muscles. Graphs done with GraphPad Prism software, San Diego, USA.

Next, we studied three relevant PKC isoforms involved in neurotransmission at the NMJ that are activated following the downstream TrkB signaling (Santafé et al., 2014; Obis et al., 2015a,b; Hurtado et al., 2017b; Simó et al., 2018, 2019). As shown in Figure 3, classical cPKCα and cPKCβI isoforms are less expressed and phosphorylated in fatigue-resistant muscles, SOL, and EOM. However, while the proportion between phosphorylated and total cPKCα detected (the phosphorylation ratio) in EOM is decreased, the ratio increases in both SOL and EOM for cPKCβI (Figures 3A,B), pointing to a differential consumption pattern through the isoforms.

On the contrary, novel nPKCε abundance is higher in SOL and EOM than in EDL and TA but it is less phosphorylated (Figure 3C). Finally, PDK1 expression, which is responsible for PKCs priming for activation (Dutil and Newton, 2000; Biondi, 2004), is sustained through the studied muscles. However, the phosphorylation ratio is lower in fatigue resistant muscles, pointing to higher consumption of them (Figure 3D).

### 3.3 Catalytic and regulatory PKA subunits are differentially expressed through muscle type

**FIGURE 4:**
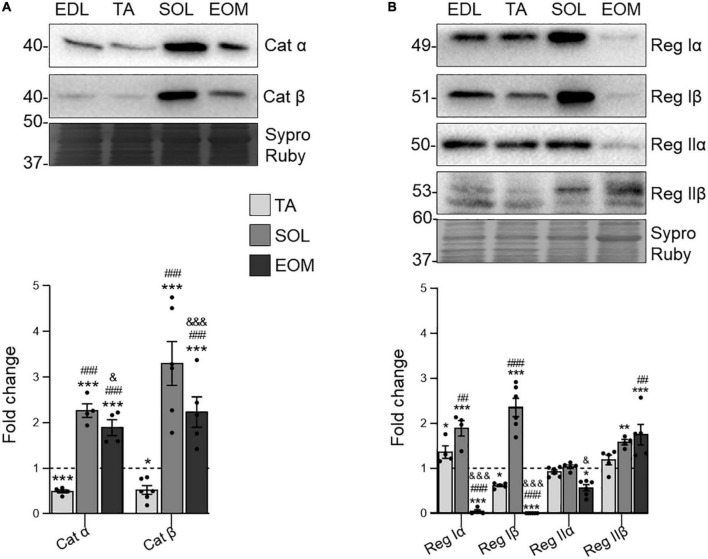
Catalytic and regulatory PKA subunits expression changes in EDL, TA, SOL, and EOM of Sprague Dawley rats. Western blot analysis and data quantification of **(A)** catalytic and **(B)** regulatory PKA isoforms protein levels in different muscles. Sypro Ruby images show the region surrounding each protein of interest despite the bands’ optical densities being normalized using the full length of the lanes. Data are presented as mean ± SEM. Each circle in the bars is the mean result of one animal. *P ≤ 0.05; **P ≤ 0.01; ***P ≤ 0.001; *against EDL, # against TA, & against SOL (Kruskal–Wallis test and Dunn’s *post-hoc* test). EDL, Extensor Digitorum Longus; TA, Tibialis Anterior; SOL, Soleus; EOM, Extraocular Muscles. Graphs done with GraphPad Prism software, San Diego, USA.

Since PKC and PKA are closely related to regulating ACh release at the NMJ (Santafé et al., 2009), we also analyzed PKA subunits in the selected muscles. The present results show that the catalytic subunits Cα and Cβ are more abundant in fatigue-resistant muscles than in fast, fatigable ones (Figure 4A). On the other hand, SOL and EOM differ in regulatory subunits expression. RI subunits are more expressed in SOL while barely detectable in EOMs, which preferentially express RII subunits (Figure 4B).

### 3.4 EOMs abundantly express the exocytotic proteins Munc18-1 and SNAP-25

**FIGURE 5:**
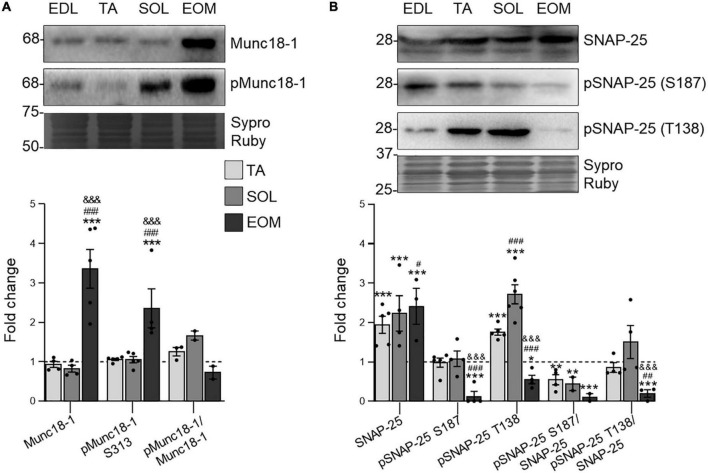
The SNARE/SM Munc18-1 and SNAP-25 expression changes in EDL, TA, SOL, and EOM of Sprague Dawley rats. Western blot analysis and data quantification of **(A)** Munc18-1 and **(B)** SNAP-25 protein levels in different muscles. Sypro Ruby images show the region surrounding each protein of interest despite the bands’ optical densities being normalized using the full length of the lanes. Data are presented as mean ± SEM. Each circle in the bars is the mean result of one animal. *P ≤ 0.05; **P ≤ 0.01; ***P ≤ 0.001; *against EDL, # against TA, & against SOL (Kruskal–Wallis test and Dunn’s *post-hoc* test). EDL, Extensor Digitorum Longus; TA, Tibialis Anterior; SOL, Soleus; EOM, Extraocular Muscles. Graphs done with GraphPad Prism software, San Diego, USA.

Both PKC and PKA phosphorylate targets are keys in the exocytosis of ACh synaptic vesicles. Munc18-1 is an essential, neuron-specific protein involved in neurotransmitter release (Südhof and Rothman, 2009), specifically expressed at the nerve terminal of the NMJ (Simó et al., 2018). The S313 phosphorylation is modulated by cPKCβI and nPKCε at the NMJ (Simó et al., 2018) and regulates Munc18-1 function (Südhof, 2013). Here, the present results show an unchanged phosphorylation ratio through the studied muscles. However, both total and phosphorylated levels of Munc18-1 are upregulated in EOM in relation to limb muscles (Figure 5A), pointing to higher requirements concerning Munc18-1 in EOM.

SNAP-25 is a component of the SNARE core complex that is essential for functional neurotransmission (Katayama et al., 2017). Thus, it is localized at the nerve terminal component of the NMJ (Simó et al., 2019). On the one hand, non-phosphorylated SNAP-25 strongly interacts with Syntaxin-1, forming the SNARE complex necessary for ACh exocytosis. On the other hand, once SNAP-25 is phosphorylated, exocytosis ends (Horváth et al., 2017) and SNAP-25 switches from forming the SNARE complex to promoting vesicle recycling and refilling, in a process that depends on PKC for the S187 residue and PKA for the T138 residue phosphorylation (Nagy et al., 2002, 2004; Leenders and Sheng, 2005). Our results show that SNAP-25 expression levels are proportional to fatigue resistance in the analyzed muscles. On the other hand, both phosphorylation residues S187 and T138 are uniquely low in EOMs. Finally, SNAP-25 S187 phosphorylation does not change through limb muscles and, therefore, the pSNAP-25 S187/SNAP-25 ratio decreases with fatigue resistance. On the other hand, pSNAP-25 T138 levels are higher in TA and SOL than in EDL and, consequently, the pSNAP-25 T138/SNAP-25 ratio does not change (Figure 5B).

## 4 Discussion

**FIGURE 6:**
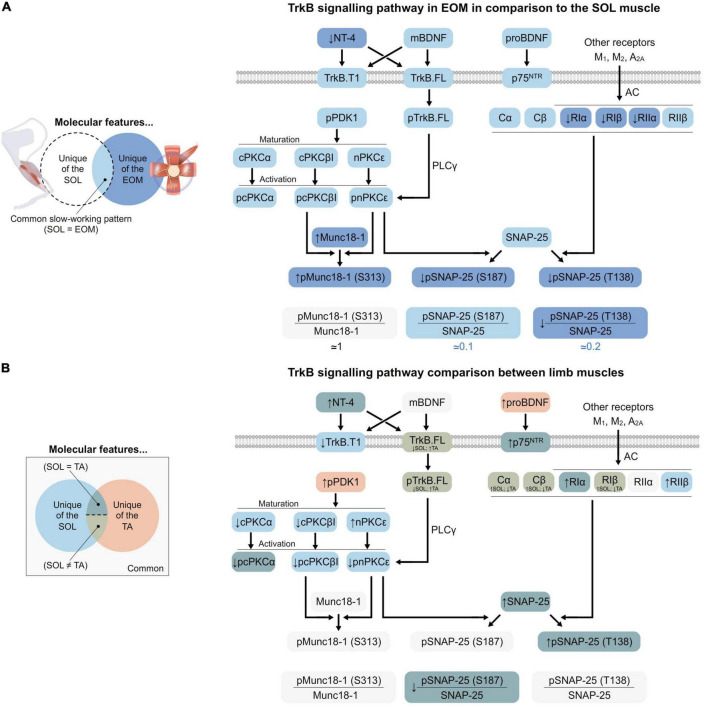
Graphical representation of the TrkB signaling pathway at the NMJ. **(A)** EOM in comparison with SOL. EOM muscles share several molecular features with SOL, represented in pale blue. However, they also have some unique features (dark blue), whose relationship with SOL is indicated by arrows (↑ for increase and ↓ for decrease). These characteristics are possibly involved in the ability of EOM to contract fast without being fatigued despite continuous usage. **(B)** The different limb muscles. Limb muscles share a few molecular features (pale gray). Also, the TA muscle has some unique features (orange). On the other hand, SOL’s unique features are always coincident with EOM (pale blue; like in panel A). Finally, some proteins equally change in SOL and TA in relation to EDL (dark green). Thus, two levels of adaptations to resistance to fatigue can be spotted depending on if they are exclusive of SOL or shared with TA. Finally, TA also shows some characteristics that could be involved in the differences between flexor and extensor muscles as they are different from SOL and EDL (pale green). The BDNF/TrkB downstream signaling is always the same at the NMJ, but the different proportions of proteins allow the adaptation to the precise requirements of each muscle and vice versa. In brief, proBDNF preferentially binds p75^NTR^ while mBDNF and NT-4 selectively bind and activate either full length or truncated TrkB. When TrkB.FL is not inhibited by heterodimerization with truncated isoforms, it activates PKCs in the membrane, which has been previously phosphorylated by PDK1. This is mediated by PLCγ, which enhances intracellular Ca^2+^ and DAG concentration. At the plasma membrane, PKCs modulate ACh release by phosphorylating exocytotic machinery proteins such as Munc18-1 and SNAP-25. Furthermore, SNAP-25 is also phosphorylated by PKA, whose activity depends on muscarinic ACh receptors, among others.

Skeletal muscles differ from one specie to the other, with activity patterns, disease conditions, and aging, among others and despite sharing functional characteristics. In consequence, skeletal muscle tissue is very plastic and its small molecular variations can induce significative differences despite being in the same generic group (i.e., fast and fatigable or slow and fatigue resistant). Until now, it was unknown whether the TrkB signaling is differently expressed in fast and slow limb muscles and in EOM, which have specific functional characteristics, resistance to fatigue, and different vulnerability to ALS neurodegenerative disease (Tjust et al., 2012). Here, we report that EOM shares molecular features of the slow fatigue-resistant soleus muscle but also particular details that correlate with EOM activity patterns. A summary of the main findings is represented in Figures 1, 6.

### 4.1 Fatigue-resistant muscles have a higher consumption of mature neurotrophic factors

NT-4 is an activity-dependent neurotrophic factor that mediates NMJ growth and remodeling (Funakoshi et al., 1995). Previous results show that in healthy fast muscles, NT-4 increases after physical exercise proportionally to the training intensity, following a fast-to-slow molecular transition (Just-Borràs et al., 2021). In accordance, the present results show that NT-4 is upregulated in the slow fatigue resistant limb muscle soleus. On the other hand, NT-4 is downregulated in EOM, which was previously related to EOM resistance to ALS (Harandi et al., 2016), pointing that NT-4 low levels in EOM could be key to confer EOMs-specific properties, understood together with neurotrophic receptor balance.

Concerning BDNF, results show a differential consumption of mBDNF among the different studied muscles, which is higher in fatigue-resistant muscles, where TrkB isoforms expression is lower. TrkB can follow the degradative pathway after binding to its ligand (Chen et al., 2005) and activating the corresponding intracellular responses. Therefore, it seems that TrkB signaling could be more intense in SOL and EOM than TrkB. FL levels are reduced, in accordance with activity-dependent consumption (González-Gutiérrez et al., 2020) and accumulated due to ALS (Just-Borràs et al., 2019). Thus, because of canonical TrkB.FL neuroprotective effect (Reichardt, 2006; Akil et al., 2016), these results correlate with EOM and SOL being more resistant to neurodegenerative diseases such as ALS, suggesting that this is an advantageous molecular phenotype at the same time that these muscles benefit from a reduced truncated TrkB expression. Indeed, the present results show that EDL and TA present higher TrkB and mBDNF levels, showing a correlation between their accumulation and decreased neuroprotective function. Indeed, these proteins are reduced in ALS-trained animals that show a better phenotype than non-trained ALS mice (Grondard et al., 2008; Just-Borràs et al., 2020).

Thus, fatigue-resistant muscles present a sustained activity of the TrkB.FL signaling pathway correlated with their stability through disease and supported by a higher TrkB.FL phosphorylation ratio in SOL and EOM. Thus, the higher TrkB.FL phosphorylation ratio observed in SOL and EOM seems to be related to the maintenance of neurotransmission. Indeed, several studies related TrkB signaling with mitochondrial function maintenance and promotion (Ahuja et al., 2022; Cheng and Lee, 2022; Li et al., 2022; Wang et al., 2022). Therefore, TrkB.FL activity seems to be proportional to mitochondrial activity, related to slower, fatigue resistant fibers. In accordance, BDNF reduction results in slower muscle metabolism and more resistance to fatigue, while BDNF accumulation induces a faster, glycolytic metabolism (Delezie et al., 2019). Altogether, mBDNF levels are proportional to the contraction speed of the muscles and inversely proportional to fatigue resistance due to consumption.

On the other hand, the neurotrophic factor receptor p75^NTR^ (Friedman and Greene, 1999) is widely expressed in the nervous system, including the NMJ (Garcia et al., 2010b), where it regulates different cellular functions depending on its interaction with co-receptors and neurotrophins (Hempstead, 2002). Particularly, during development, proBDNF promotes synaptic plasticity and refinement through p75^NTR^ (Garcia et al., 2011). Similarly, exercise promotes p75^NTR^-mediated plasticity mechanisms to refine and optimize mature adult NMJ connections (Eslami et al., 2018; Zhan et al., 2018). Indeed, mature NMJ in p75^NTR–/–^ mice showed impaired structural complexity correlated with altered synaptic function and increased susceptibility to fatigue (Pérez et al., 2019). These results correlate with decreased p75^NTR^ expression in fast plantaris muscles of ALS mice (Just-Borràs et al., 2019) while it is maintained in the EOMs despite ALS (Harandi et al., 2016). Furthermore, exercise recovers p75^NTR^ in the limb muscles of these animals (Just-Borràs et al., 2020). In accordance, the present results show that p75^NTR^ levels are proportional to fatigue resistance. Indeed, p75^NTR^ is upregulated in EOM myogenic progenitor cells, in relation to higher proliferative and fusion rates and better regenerative properties (Carrero-Rojas et al., 2020). Thus, p75^NTR^ seems to be related to selective muscle resistance.

### 4.2 PKC isoforms differential expression identifies myocyte metabolic profile

As a result of TrkB.FL-mediated PLCγ1 activation, mature PKCs are anchored to the membrane (Colón-González and Kazanietz, 2006; Griner and Kazanietz, 2007). PKC maturation and correct folding require a priming phosphorylation step by PDK1 (Dutil and Newton, 2000; Biondi, 2004), which is promoted by synaptic activity at the NMJ (Hurtado et al., 2017b). In mammalian NMJ, PDK1 is self-phosphorylated in the activation loop residue S241 to be constitutively active (Casamayor et al., 1999; Hurtado et al., 2017a). The present results show a decrease in phosphorylated PDK1 (S241) in EOMs, which could be related to consumption and degradation after its function (Hurtado et al., 2017a), pointing to more intense activity in relation to the studied limb muscles.

Once PKCs are phosphorylated by PDK1, they can sense intracellular second messengers and bind to the membrane for substrate phosphorylation (Colón-González and Kazanietz, 2006). Afterward, PKCs are degraded by the proteasome in an activation-dependent manner (Gould and Newton, 2008). Studies have proposed that classic cPKC might be activated by fast-twitching patterns of activity (Pedersen et al., 2009). Here, we add new evidence that cPKCα and cPKCβI are reduced in fatigue-resistant muscles. Thus, in rodents, like in avian models (Gundersen, 2011), cPKC activity is higher in fatigable muscles than in fatigue-resistant ones. Indeed, cPKCα elevation was correlated with downregulated slow MHC isoforms in slow muscles (DiMario, 2001). In accordance, the ratio of phosphorylated cPKCβI in fatigue-resistant muscles triplicates the one in fatigable muscles, which could be understood as an accumulation, showing that cPKCβI is less active in SOL and EOM than in TA and EDL. These differences are not evident for cPKCα, probably in relation to its cellular distribution, as cPKCα is ubiquitously located while cPKCβI is confined to the presynaptic component (Besalduch et al., 2010).

On the other hand, novel nPKC isoforms are more active in slow fibers (Yuan et al., 2002; Rykx et al., 2003) in correlation with increased myoglobin levels, reporting a slower metabolism in these muscles (Kim et al., 2008). In the present results, nPKCε abundance, which is only expressed in the presynaptic component (Obis et al., 2015a) is accompanied by pnPKCε consumption in SOL and EOM, which could be correlated with constant and higher activity of this isoform guaranteed by a pool of available protein. Altogether SOL and EOM share PKC expression levels, being a potential reason for the resistance to fatigue in these muscles (Santafé et al., 2009; Hurtado et al., 2017b; Simó et al., 2018, 2019). On the other hand, TA and EDL also share PKC expression levels, making a strong difference between fast, fatigable, and fatigue-resistant muscles. Thus, in accordance with previous results (Gundersen, 2011), and due to their exclusive presence in the presynaptic terminal, cPKCβI and nPKCε (Besalduch et al., 2010; Obis et al., 2015a) could be considered as candidates to identify fast and slow NMJ in limb muscles, respectively.

### 4.3 PKA activity is higher in fatigue resistance than in fast fatigable muscles

cAMP-dependent kinase regulatory isoforms have a specific tissue and subcellular distribution (Colledge and Scott, 1999). RIα and RIIα are widely expressed through tissues; RIβ is highly expressed in the nervous tissue and RIIβ is especially abundant in adipose and hepatic tissues (Cadd and McKnight, 1989; Brandon et al., 1997; Skalhegg and Tasken, 2000; Fagerberg et al., 2014). As previously reported (Hoover et al., 2001), our results show that PKA subunits are always higher in SOL than EDL, probably in relation to the functional differences between fast and slow muscles. In addition, following RI major affinity to release catalytic subunits, their scarcity in EOM could indicate higher PKA activity in EOM than in SOL. On the other hand, RIIβ levels were proportional to fatigue resistance, which correlates with RIIβ expression increase in the fast plantaris after endurance training due to a fast-to-slow molecular transition (Just-Borràs et al., 2021). Furthermore, slow fibers have been reported to accumulate more lipids than fast fibers with age in human subjects (Gueugneau et al., 2015; Choi et al., 2016), which could explain the prevalence of RIIβ in EOM and SOL since RIIβ is abundant in the adipose tissue (Cadd and McKnight, 1989; Brandon et al., 1997; Skalhegg and Tasken, 2000; Fagerberg et al., 2014), maybe in association with the lipids that are in there.

### 4.4 EOM fatigue-resistant muscles show increased phosphorylation and synthesis of Munc18-1 and SNAP-25 exocytotic proteins

PKCs phosphorylate several proteins that are essential for the exocytotic mechanism. On the one hand, Munc18-1, which favors membrane fusion when associated with the SNARE complex (Südhof, 2013), depends on two PKC phosphorylation sites S306 and S313 (Fujita et al., 1996; Barclay et al., 2003; Morgan et al., 2004; Snyder et al., 2006). Particularly, at the NMJ, S313 phosphorylation depends on the coordinated action of presynaptic nPKCε and cPKCβI and the retrograde TrkB signaling (Simó et al., 2018).

The present results show that Munc18-1 and pMunc18-1 levels are higher in EOM than in EDL, TA, and SOL. However, despite PKC activity differences, the Munc18-1 phosphorylation ratio is maintained through the four muscles. In SOD1-G93A mice, the amount of Munc18-1 able to interact with the SNARE complex is reduced due to over-phosphorylation (Just-Borràs et al., 2019) and in relation to neurotransmission failure (Eisen, 2001; Rocha et al., 2013). On the other hand, exercise normalizes pMunc18-1 accumulation in ALS mice (Just-Borràs et al., 2020), in correlation with an improvement of the NMJ function (Genç et al., 2014) evidenced by a better motor function (Deforges et al., 2009). Thus, the maintenance of the pMunc18-1/Munc18-1 ratio through muscles, as in trained ALS mice, seems to be essential for a correct neuromuscular function. Furthermore, the high availability in the EOM could be related to the specific release properties of their MNs.

On the other hand, SNAP-25, a member of the exocytotic SNARE complex, is phosphorylated by PKC at S187 in response to synaptic activity and elevated intracellular calcium levels, which induces its translocation to the membrane. At the NMJ, nPKCε mediates this process especially during synaptic activity, while muscle contraction exerts negative feedback to return S187 phosphorylation to basal levels (Simó et al., 2019). In particular, pSNAP-25 S187 negatively modulates neuronal activity during intense activation (Verderio et al., 2004; Pozzi et al., 2008). Consequently, the absence of SNAP-25 blocks fast calcium-triggered exocytosis, as the primed pool of synaptic vesicles becomes empty. This is in accordance with S187 phosphorylation downregulation resulting in decreased vesicle secretion (Sørensen et al., 2002, 2003) while phosphorylated S187 mutant overexpression accelerates vesicle recruitment after the emptying of the releasable vesicle pool (Nagy et al., 2002). Here, results show that SNAP-25 total levels are upregulated in all but EDL muscle, indicating that it could be a limiting factor for continuous neurotransmission in accordance with its function. Surprisingly, S187 phosphorylation is uniquely very low in EOMs, which could be understood as a result of continued consumption that is guaranteed by nPKCε availability. In accordance, the SNAP-25 S187/SNAP-25 ratio gradually decreases, being inversely proportional to fatigue resistance.

On the other hand, SNAP-25 T138 is phosphorylated by PKA (Hepp et al., 2002). This phosphorylation is not essential for SNARE complex assembly and stability (Risinger and Bennett, 1999) but it is necessary to maintain the ready releasable and primed pool of vesicles (Nagy et al., 2004). Here, results show that the pSNAP-25 T138/SNAP-25 ratio only decreases in EOM. Furthermore, this occurs in parallel with low pSNAP-25 S187 in EOMs, which makes us hypothesize that SNAP-25 phosphorylated residues are quickly lost after their action which could be related to their non-stop activity. Thus, the exceptionally low level of pSNAP-25 (both S187 and T138) in the EOM may facilitate the SNARE-SM complex formation, stability, and vesicle docking. Indeed, in opposition to healthy conditions, both pSNAP-25 S187 and T138 and total SNAP-25 were accumulated in ALS mice (Just-Borràs et al., 2019), in correlation with neurotransmission failure, showing that SNAP-25 was not able to work properly for exocytosis. These, together with high pMunc18-1 levels point to a singular regulation of the vesicular release mechanism specially related to phosphorylation residues conservation time.

## 5 Concluding remarks

The TrkB signaling is crucial for the correct bidirectional communication between the MN and the muscle fibers that they innervate (Hurtado et al., 2017b; Simó et al., 2018, 2019). This allows a precise functionality of the NMJ and, therefore, the contractibility of the muscle. Here, the present results show that healthy muscles have an individualized molecular pattern of the TrkB pathway, involved in the neurotransmission, that guarantee the achievement of their precise functions even despite the fact of having very similar contraction velocities and fatigue resistance, such as TA and EDL. In brief, TrkB signaling seems to be more active in slow than in fast muscles, in accordance with higher neurotrophic factors consumption and following their resistance to fatigue and vulnerability to neurodegenerative diseases such as ALS. In accordance, BDNF reduction results in slower muscle metabolism and more resistance to fatigue, while its accumulation is related to a faster metabolism (Delezie et al., 2019). Furthermore, these differences allow us to understand and differentiate the phenotype of healthy muscles and their susceptibility to changes in activity patterns. Consequently, the relation between the synaptic and neurotrophic control at the NMJ is adapted to the precise characteristics of each motor unit. EOMs are of particular interest as they have distinctive characteristics that guarantee fast contraction but high resistance to fatigue and remarkable resistance to neurodegenerative diseases such as ALS. Here, we show that wild-type EOM has a specific molecular phenotype of the TrkB signaling pathway, including enhanced retrograde neurotrophic control and more robust machinery to maintain constant synaptic activity oriented to provide a slow performance of the muscles without compromising resistance to continuous usage, summarized in Figure 6.

## Data availability statement

The raw data supporting the conclusions of this article will be made available by the authors, upon reasonable request.

## Ethics statement

The animal study was reviewed and approved by Ethics Committee of Animal Experimentation of the Universitat Rovira i Virgili.

## Author contributions

LJ-B: data collection and quantitative analysis and statistics. VC-M: graphical abstract design. LJ-B, JT, ML, and NG: literature search. LJ-B, JT, and ML: manuscript preparation. JT and ML: conception and design. All authors data interpretation, read, and approved the final manuscript.
